# Strain Characterisation for Measuring Bioefficacy of ITNs Treated with Two Active Ingredients (Dual-AI ITNs): Developing a Robust Protocol by Building Consensus

**DOI:** 10.3390/insects13050434

**Published:** 2022-05-06

**Authors:** Rosemary S. Lees, Jennifer S. Armistead, Salum Azizi, Edi Constant, Christen Fornadel, John E. Gimnig, Janet Hemingway, Daniel Impoinvil, Seth R. Irish, William Kisinza, Natalie Lissenden, Henry D. Mawejje, Louisa A. Messenger, Sarah Moore, Corine Ngufor, Richard Oxborough, Natacha Protopopoff, Hilary Ranson, Graham Small, Joseph Wagman, David Weetman, Sarah Zohdy, Angus Spiers

**Affiliations:** 1 Department of Vector Biology, Liverpool School of Tropical Medicine, Pembroke Place, Liverpool L3 5QA, UK; janet.hemingway@lstmed.ac.uk (J.H.); natalie.lissenden@lstmed.ac.uk (N.L.); hilary.ranson@lstmed.ac.uk (H.R.); david.weetman@lstmed.ac.uk (D.W.); 2 Innovation to Impact, Pembroke Place, Liverpool L3 5QA, UK; angus.spiers@innovation2impact.org; 3 U.S. President’s Malaria Initiative (PMI), U.S. Agency for International Development (USAID), Washington, DC 20547, USA; jarmistead@usaid.gov; 4 KCMUCo-PAMVERC Test Facility, Department of Medical Parasitology and Entomology, Kilimanjaro Christian Medical University College, Moshi P.O. Box 2240, Tanzania; salum.azizi@pamverc.or.tz; 5 Centre Suisse de Recherches Scientifiques (CSRS), Abidjan 1303, Côte d’Ivoire; constant.edi@csrs.ci; 6 Innovative Vector Control Consortium (IVCC), Liverpool School of Tropical Medicine, Liverpool L3 5QA, UK; christen.fornadel@ivcc.com (C.F.); graham.small@ivcc.com (G.S.); 7 Division of Parasitic Diseases and Malaria, Centers for Disease Control (CDC) and Prevention, Atlanta, GA 30329, USA; hzg1@cdc.gov (J.E.G.); xda6@cdc.gov (D.I.); ykr2@cdc.gov (S.Z.); 8 U.S. President’s Malaria Initiative (PMI), Centers for Disease Control (CDC) and Prevention, Atlanta, GA 30329, USA; seth.irish@swisstph.ch; 9 Amani Research Centre, National Institute for Medical Research, Muheza P.O. Box 81, Tanzania; wnkisinza@gmail.com; 10 Infectious Diseases Research Collaboration (IDRC), Plot 2C Nakasero Hill Road, Kampala P.O. Box 7475, Uganda; mawejjehenry@yahoo.com; 11 Department of Disease Control, Faculty of Infectious Tropical Diseases, London School of Hygiene and Tropical Medicine, Keppel Street, London WC1E 7HT, UK; louisa.messenger@lshtm.ac.uk (L.A.M.); corine.ngufor@lshtm.ac.uk (C.N.); natacha.protopopoff@lshtm.ac.uk (N.P.); 12 Vector Control Product Testing Unit (VCPTU), Environmental Health and Ecological Science Department, Ifakara Health Institute, Bagamoyo P.O. Box 74, Tanzania; smoore@ihi.or.tz; 13 Vector Biology Unit, Department of Epidemiology and Public Health, Swiss Tropical & Public Health Institute, Kreuzstrasse 2, Allschwil, 4123 Basel, Switzerland; 14 Faculty of Science, University of Basel, Petersplatz 1, 4001 Basel, Switzerland; 15 Nelson Mandela African Institute of Science and Technology (NM-AIST), Tengeru P.O. Box 447, Tanzania; 16 Centre de Recherche Entomologique de Cotonou, Cotonou BP 2604, Benin; 17 PMI VectorLink Project, Abt Associates, 6130 Executive Blvd., Rockville, MD 20852, USA; richard_oxborough@abtassoc.com; 18 Malaria and Neglected Tropical Diseases Program, PATH, Washington, DC 20001, USA; jwagman@path.org

**Keywords:** insecticide-treated nets (ITN), pyrethroid, mosquito, strain characterisation, insecticide resistance, method development, durability monitoring, product evaluation, quality control (QC), dual active ingredients (dual-AI), bioefficacy

## Abstract

**Simple Summary:**

New types of bed nets are being developed which contain a pyrethroid plus a second chemical because of the development and increased frequency of mosquito mechanisms to avoid being killed by pyrethroids. When insecticide-treated bed nets are being trialed for efficacy or released onto the market it is essential to measure how effective the net is in killing mosquitoes, which includes testing how quickly insecticide is lost or degraded due to routine wear and tear. Pyrethroid-resistant mosquitoes are needed to test the effectiveness and insecticidal persistence of the second chemical, because they are not all killed by the pyrethroid, allowing the killing effect of the two chemicals to be evaluated independently. However, because resistance status varies between populations of mosquitoes that possess different resistance mechanisms, and because resistance intensity in a population can change over time, a method is needed to characterise the resistant mosquitoes. A focus group of experts discussed how this should best be done, considering pros and cons of different approaches, and co-wrote a protocol, which will be valuable for malaria control programmes and stakeholders wanting to test the effective lifespan of a new bed net in terms of the active ingredient bioefficacy.

**Abstract:**

Durability monitoring of insecticide-treated nets (ITNs) containing a pyrethroid in combination with a second active ingredient (AI) must be adapted so that the insecticidal bioefficacy of each AI can be monitored independently. An effective way to do this is to measure rapid knock down of a pyrethroid-susceptible strain of mosquitoes to assess the bioefficacy of the pyrethroid component and to use a pyrethroid-resistant strain to measure the bioefficacy of the second ingredient. To allow robust comparison of results across tests within and between test facilities, and over time, protocols for bioefficacy testing must include either characterisation of the resistant strain, standardisation of the mosquitoes used for bioassays, or a combination of the two. Through a series of virtual meetings, key stakeholders and practitioners explored different approaches to achieving these goals. Via an iterative process we decided on the preferred approach and produced a protocol consisting of characterising mosquitoes used for bioefficacy testing before and after a round of bioassays, for example at each time point in a durability monitoring study. We present the final protocol and justify our approach to establishing a standard methodology for durability monitoring of ITNs containing pyrethroid and a second AI.

## 1. Introduction

Insecticide-treated nets (ITNs) have been critical in controlling malaria. However, widespread resistance to the pyrethroids, which have been the sole insecticide class used on all ITNs until recently, threatens the continued effectiveness of standard ITNs [1]. Therefore, there is a need for new ITNs that include insecticides from classes with new modes of action to combat pyrethroid-resistant vector populations [2]. Several ITNs have been pre-qualified by the World Health Organization (WHO) containing a pyrethroid plus a second active ingredient (AI), which may be another insecticide (chlorfenapyr, CFPR; pyriproxyfen, PPF) or the synergist piperonyl butoxide (PBO), hereafter referred to as dual-AI ITNs [3].

There is a need to test the bioefficacy of ITNs in the laboratory. Here we are using the term ‘bioefficacy’ to mean the ability of a net sample to kill mosquitoes in a bioassay, contrasted with the efficacy of an ITN, which describes the net’s ability to meet its objective of offering personal and community protection against transmission of mosquito-borne disease. Prototypes may need to be compared during product development, and research may be conducted to explore how an ITN works. Before distributing an ITN, the national malaria control programmes (NMCPs), or funders, may want to test its efficacy against local mosquito populations. During randomised control trials to determine the efficacy of ITNs (for example [4,5]), and during post-deployment monitoring (for example [6]), use and attrition of ITNs are monitored, and samples of deployed nets are collected over time to monitor their physical durability, analyse insecticide content and measure the bioavailability of each AI, using agreed-upon and validated bioassay methodologies (i.e., WHO cone bioassay or tunnel tests) [7,8]. This testing may be done at the time of collection, or all samples may be accumulated for simultaneous testing at the end of the study. Existing methods for durability monitoring [9], are optimised to evaluate pyrethroid-only ITNs, but the bioassay component may need to be adapted to be suitable for dual-AI ITNs.

The ability of a dual-AI ITN to kill insecticide-susceptible mosquitoes can be measured using standard methodologies and a susceptible laboratory strain. If the entomological endpoint of the second AI is different to the rapid knockdown and kill achieved by a pyrethroid, it will be possible to separate the effect of the pyrethroid and the second AI. To monitor the persistence and additional efficacy of the second AI, a pyrethroid-resistant strain must be used, the majority of which will survive contact with the pyrethroid so that the effect of the second AI can be measured. Traditionally, mortality caused by pyrethroid exposure is measured to 24 h, as this insecticide class is fast acting. To control for delayed mortality caused by the pyrethroid in a resistant strain, where the second AI causes delayed mortality, mortality could be measured to the same time point when characterising pyrethroid susceptibility. The nature of the resistant strain needs to be considered, as this will affect the interpretation of data from the durability monitoring testing. The Vector Control Advisory Group (VCAG) of the WHO proposed the following criteria, in 2014, for mosquito strains suitable for use in screening for cross-resistance between insecticidal products [10]: at least 3 strains, two of which have significant metabolic resistance, representing the broad spectrum of known resistance mechanisms, ideally from a provided list of standard strains, or a strain that is fully characterised, and has a resistance level greater than 10-fold that of a susceptible strain of the same species at the LC_50_, tested in parallel. Though not specified at the time, this LC_50_ would ideally be measured at the time point of interest for the second AI. This may form the basis of selecting suitable strains for bioefficacy testing or durability monitoring of dual-AI ITNs, but developments in the understanding of the molecular characteristics of mosquitoes have been made since these recommendations were released. New modes of action of insecticide are now being considered, meaning that there are limitations to these criteria, and practical challenges in meeting them, and they should, therefore, be reviewed. 

The number of different resistance mechanisms that have now been identified, and would need to be screened to characterise a strain fully, is increasing over time and include overexpression of detoxifying enzymes [11,12], involvement of sensory appendage proteins [13] or the salivary protein gland [14,15], or cuticular thickening [16,17]. Insecticide resistant mosquito populations possess different combinations of mechanisms, and the relative contribution of these mechanisms to resistance differs between populations. These features have evolved to confer resistance to insecticides to which mosquitoes have been exposed, but some may also confer cross-resistance to new insecticides even with novel modes of action. Representing all known resistance mechanisms even in three strains, would be a major challenge, and, given that both the range of mechanisms expressed and our knowledge of these evolves over time, will always risk omitting resistance mechanisms that have not yet been identified. This is of particular concern for entirely new AIs coming to market, resistance mechanisms for which have not been identified.

Even if a list of standard representative laboratory colonies was established, there would be no guarantee of expected results in testing between sites or across time. For example, a colony that is nominally from a common strain may differ from a colony of the same strain held at a different test facility, due to differences in establishment and/or stabilisation in new laboratories and related selection pressures, genetic drift, inbreeding and genetic bottlenecks [18], insecticide exposure to maintain resistance [19] or through contamination events, rearing conditions that may affect fitness [20,21,22] or microbiome characteristics [23,24]. Resistance may shift over time, particularly if a strain is transferred between facilities or if selection pressure is not maintained. In addition, mosquito strains show temporal variability in their physiological response to insecticides. Routine efforts to characterise resistance phenotypes in lab strains are commonly based on the use of discriminating concentrations (DCs) or resistance intensity assays, rather than dose-response assays, which would be needed to establish LC_50_ values and resistance ratios. 

Modes of action of insecticides used in dual-AI ITNs currently under evaluation mean that bioassays and protocols designed to measure bioefficacy of a pyrethroid may not be suitable. When considering chlorfenapyr, for example, the metabolic status of a mosquito is believed to affect metabolism of the pro-insecticide to the active form, and subsequently mortality rate [25]. Metabolic rate may be affected by extrinsic factors, such as temperature, time of day [26], and intrinsic factors, such as the nutritional status of the mosquito [27,28,29]. Bioassay choice and design may play a part, affecting, for example, how much mosquitoes move, or whether mosquitoes blood feed. As well as having higher rates of expression of detoxifying enzymes, different strains may have different metabolic rates, which may be related to the resistance mechanisms they express. 

Finally, different criteria may apply when selecting a single strain or multiple strains to monitor the bioefficacy of dual-AI ITNs for durability monitoring, which is the focus of the present study. For example, to monitor the durability of ITNs it is not critical that the target species is used for bioefficacy testing, as long as the sensitivity of the species used is such that the bioefficacy of each AI can be detected across a relevant concentration range. 

When conducting a durability study on ITNs, testing all samples at a similar time at the end of the study may help to minimise any temporal rearing effects on mosquito strains. Alternatively, testing ITN samples as they are collected avoids the logistical resources needed to store nets or net samples, and will minimise net degradation and/or loss of insecticide bioavailability before the bioassays. However, correct storage, according to manufacturers’ instructions, should minimise degradation. Regardless of the approach, the large number of samples means it will not be possible for all net samples to be assayed by the same facility, at the same time, using the same cohort of resistant mosquitoes. In order to be able to compile and compare results of testing across a study, between facilities and time points, and to compare results between studies, there is a requirement for either (a) standardisation, such that the method and inputs are the same in all cases to minimise differences between results, or (b) characterisation of the inputs, so that results can be interpreted and, where differences are seen, any disparities between the inputs can be taken into account, or (c) a combination of the two. Depending on the specific questions of a study it might not be interesting to separate out the bioefficacy of each AI in a dual-AI ITN, and it may be sufficient to test the relevant endpoints in the mosquito population of interest or to compare results of bioefficacy testing with chemical analysis results on the same net samples. For the purpose of this consultation, we were interested in being able to separate out the effects of each AI, which is particularly relevant for randomised control trials (RCTs) of new types of ITN, where the durability of the second AI may not be known but is important to understand the added benefit over a pyrethroid-only ITN. In this case, the pyrethroid may be tested using a standard susceptible strain of the target species in the case where the second AI has an effect other than the rapid knock down and mortality caused by pyrethroids, but to test the additional benefit of the second AI (chlorfenapyr (CFPR), pyriproxyfen (PPF) or piperonyl butoxide (PBO)) mosquitoes must be pyrethroid-resistant, and assessed over the timescale of action relevant to the second AI. Inputs to the protocol for durability monitoring of dual-AI ITNs, therefore, include the pyrethroid-resistant mosquitoes used to test the second AI. 

Selection, characterisation and standardisation of resistant mosquitoes are complex. The consultation process described in this paper aimed to produce a guide to the use of resistant strains for laboratory bioefficacy testing of dual-AI ITNs. Bioefficacy testing of ITNs tests for the presence of sufficient quantities of bioavailable compound to induce the desired endpoint in mosquitoes, usually mortality, and repeated testing over time can be used to measure durability of an ITN, for example, during an RCT trial. The purpose of this consultation was to develop, by consensus, a protocol for ensuring that the use of pyrethroid-resistant mosquitoes can be sufficiently characterised or standardised to allow compilation, comparison and interpretation of bioefficacy data across studies designed to monitor durability. A standard operating procedure (SOP) was produced which can be used by project teams, and is a supporting document to consensus SOPs recently developed for durability monitoring of new net types [30]. The same SOP may be useful in characterising a pyrethroid-resistant strain of mosquitoes for other purposes, or it could be adapted to meet different specific needs. This project forms part of a package of work to improve entomological methods in vector control, and is supported by Innovation to Impact (I2I) at the Liverpool School of Tropical Medicine (LSTM). 

## 2. Materials and Methods

A group of experts was assembled, based on attendance at a preliminary discussion during a sidebar meeting at the American Society of Tropical Medicine and Hygiene (ASTMH) conference in November 2019, with additional invitees identified by the initial group, based on research interest in insecticide resistance, experience in the testing of new net types, or involvement in current or recent community scale trials of ITNs. Four virtual meetings of these stakeholders were held between April 2020 and August 2021, during which the need for a means to standardise or characterise resistant mosquitoes for the purpose of efficacy testing of dual-AI ITNs was agreed upon, possible approaches proposed and advantages and challenges of each discussed. Based on these discussions a protocol was drafted and iteratively refined by the group, who all then approved the final consensus SOP. A summary of these discussions, the final protocol, and the justifications for arriving at the proposed approach are presented here, and a detailed SOP is included as Supplementary Information. 

## 3. Results

### 3.1. Possible Approaches to Achieve Standardisation or Characterisation

Several approaches to achieve either standardisation or characterisation were considered, outlined in Table 1. The advantages and disadvantages of each approach were discussed before a consensus approach was developed (Section 3.2).

#### 3.1.1. Distribute the Same Well-Characterised Resistant and Susceptible Strains to All Test Facilities

Where bioassays need to be carried out at multiple facilities, one approach could be distributing a suitable resistant and susceptible strain to all facilities. Robust characterisation in the originating centre, and suitable quality control measures in receiving facilities, should remove strain differences as a variable in the assay. Strains could be maintained under the same selection and profiling regime, refreshed from a single facility if results of regular profiling start to differ, or refreshed every few generations from a single facility. 

There are some practical limitations to this approach. The nature of a suitable strain may differ depending on the second AI under evaluation, and so this exercise may need to be repeated in parallel for each dual-AI ITN in a study. There may also be little benefit in terms of the workload of this approach over others. In the longer term, there are benefits to building the capacity of facilities to establish, maintain and characterise local strains. Regardless, this is the most straightforward approach to standardisation, provided the maintenance of the strain could be standardised between facilities.

However, there are two insurmountable issues. Firstly, it is unlikely that the same strain distributed and maintained in different facilities will remain static and comparable regarding its resistance profile. Even when under continued selective pressure, resistant phenotypes can shift over time [19,31,32], and there is likely to be a change in the resistance profile of strains associated with different genetic bottlenecks when moving between facilities, both as a result of small founding populations and as colonies adapt to their new environment. Even when rearing and selection are done under the same laboratory conditions, potential supplementary factors which are gaining more attention, such as the mosquito microbiome, may differ between insectaries and affect tolerance to insecticides [33,34,35,36]. Genetic drift will occur over time, even in well-controlled rearing facilities like the Malaria Research and Reference Reagent Resource Centre (MR4) at BEI Resources [37]. Changes in resistance can occur quite quickly [38]. So important differences may be missed before testing if the strain was not refreshed or re-characterised regularly to confirm that the resistance phenotype was still as expected. 

Second, and perhaps most importantly, because of the biohazard risk inherent in transferring resistant mosquitoes between geographical regions, there are strong reservations concerning this approach. There would need to be strict containment and quality control measures in place in all receiving facilities, but even then, there is a major ethical consideration in moving a strain that is potentially more resistant than the wild populations surrounding the second test facility. The MR4, for example, will perform case-by-case hazard assessments before distributing Anopheles strains and would not distribute strains where there is a risk of laboratory/insectary escape and potential for introduction establishment of a novel resistant population in a new environment [37]. In some situations, relevant parties may accept the idea, but the containment measures needed to make this approach safe may be too expensive or not feasible in practice. Alternatively, national, local or facility decision-makers may refuse to take on this responsibility and receive the mosquito strains. For these reasons, this is not a practical approach.

#### 3.1.2. Each Testing Facility Uses Its Own Characterised Resistant Strain with a Single Standardised Protocol

Before a trial begins, the bioassay methodology used for bioefficacy testing, for example, as part of durability monitoring, should be optimised and validated using new and twenty times washed dual-AI ITNs with a susceptible strain (e.g., Kisumu). An additional validation step could be added with a range of different well-characterised pyrethroid-resistant strains to demonstrate that the method is not sensitive to differences in resistance mechanisms or population differences. In this context, a well-characterised strain would be one for which the phenotypic resistance profile was known, ideally with some understanding of the target site mutations, level of expression of detoxifying enzymes and other known mechanisms. Most test facilities that perform durability monitoring already hold pyrethroid-resistant strains that take some effort to characterise. They will often maintain them under selective pressure to preserve the resistant phenotype. There is a growing desire in the community to increase the capacity of local institutions, so this will increasingly be the case. Therefore, a pragmatic approach to standardisation could be that each facility uses a characterised local strain and relies on the testing methodology’s robustness to give consistent results between facilities. This might be an attractive option to National Malaria Control Programmes (NMCPs) that would like to see data against mosquitoes that are closest in phenotype and genotype to the local mosquitoes that are responsible for malaria transmission.

If this approach were to be adopted as a way to compare results between sites, the method would need to be tested against a sufficient number of genetically heterogeneous strains, which would need to be sufficiently different for the validation to provide convincing evidence that the results would be comparable no matter what strain was used. There is some precedent. The WHO’s VCAG have suggested three strains be used to screen for cross-resistance [10]. Phase II efficacy trials of ITNs require testing in an area with mosquitoes susceptible to all compounds in the ITN under evaluation, followed by testing in an area with pyrethroid-resistant populations [39]. However, it is unclear whether testing a single sample of nets against three resistant strains would provide sufficient evidence. This would be a significant burden in the efforts to validate a method. Assuming that containment facilities are not available, a single test facility is unlikely to have strains covering a broad geographical range. It is also unlikely that all resistance mechanisms will be represented by the strains the facilities maintain, thus necessitating a multi-centre validation approach.

This solution assumes that the testing methodology is sufficiently robust and specific enough and that the pyrethroid resistance is sufficiently high in the strains used that it is truly a test of the efficacy of the second AI alone. Since evidence for differences in resistance levels within the class is weak [40], characterising resistance to one example pyrethroid, or perhaps one representative Type I and one Type II pyrethroid, would be sufficient. However, even with very resistant strains, some individuals are usually killed by exposure to pyrethroids, and mortality can vary substantially within, and between, bioassays [40]. So, some measure of the additional impact of the second AI is likely to be still needed, for example, a comparison to a pyrethroid-only ITN. 

The group did not have confidence in comparability between data collected at different facilities with different strains. A bioassay is not likely to be validated sufficiently to give the same results, no matter the strain used for testing, because, for the following reasons, characterisation of strains will not be perfect: not all resistance mechanisms have been identified; those contributing most to resistance are not well understood; and not all markers are routinely screened for in all test facilities. There is evidence of this challenge in efforts by the WHO to set DCs for AIs by testing compounds against multiple strains of the same species and selecting a suitable dose based on the consensus of data [41]. The consensus opinion was that, although this is a pragmatic solution, the use of different strains with different resistance mechanisms and rearing methods are unlikely to give consistent results between test facilities or across time points, and so this was not the preferred option.

#### 3.1.3. Characterisation of the Resistant Strain in Parallel to the Durability Monitoring Testing

The resistance phenotype of mosquitoes used for bioefficacy testing of dual-AI ITNs could be characterised by the following to ensure that they are suitable to effectively provide the information needed: sufficient resistance to pyrethroids, such that a high enough proportion survive exposure to the pyrethroid that the effects of the second AI can be measured, and susceptibility to the second AI. WHO tube bioassays to assess the susceptibility of the proposed strain to the WHO DC [42] of the pyrethroid, as well as the second AI included on the dual-AI ITN under evaluation, where a DC and method for evaluation are available, would be appropriate for this purpose; a straightforward and familiar method. Resistance intensity or dose-response assays with the AIs of interest would provide some quantitative information to help in defining a strain. A clear definition of a strain suitable for use in testing would be required, and the rejection criteria would need to offer a balance between pragmatism and the need for robust results. 

Further characterisation could be done to further understand the strain and aid in the interpretation of results. This would require clear guidance on interpreting the bioassay results in the context of the strain characterisation. These could include, for example, DC assays with examples of type I and type II pyrethroids. All locally used insecticide classes in use for mosquito control could more fully characterise the strain’s resistance profile. Testing for the presence of molecular markers associated with insecticide resistance would be informative, if the most informative or relevant molecular markers could be determined. This may not be practical on a routine basis, but strains held for bioefficacy testing would ideally be regularly screened for key molecular markers to provide a background understanding of the resistance profile of a strain and interpretation of data, e.g., response to PBO. In order to predict the efficacy of the product under evaluation, it would be helpful to confirm that a strain possessed key resistance mechanisms, against which the product under evaluation claims efficacy, and susceptibility to the second AI.

Beyond the resistance phenotype, there are multiple sources of variability between bioefficacy tests related to the mosquitoes used. Biological factors can affect observed levels of insecticide resistance, which may lead to differences between cohorts of mosquitoes from the same strain. For example, size [27], nutritional status [27,43,44], the temperature during rearing [28,45], and age [46,47], can all have an effect on mosquito fitness, and conditions during testing affect the results of bioassays [46] and references therein. Routine quality control and use of rearing SOPs (e.g., [19,48,49]) would be a robust method of ensuring that suitable mosquitoes are used throughout the study and across facilities and would ideally include fitness testing as a measure of the consistency of rearing methods and quality of the adults produced. When maintained in the absence of selective pressure, or selective pressure only from a single insecticide, resistance phenotypes and genotypes can shift in a laboratory colony over time [19]; regular selection for insecticide resistance should form part of a programme of quality control in maintaining a resistant strain of mosquitoes. If a strain is intended for pyrethroid testing, it should be selected using a pyrethroid only insecticide, whereas a multi-resistant strain could be periodically selected with different insecticide classes, though this would be a significant undertaking.

While good rearing and testing procedures minimise most sources of variation [50], it would be informative to include some fitness testing (for example, wing length, average weight) of a sample of the cohort of mosquitoes used for bioefficacy testing, or, if possible, a sample from individuals which were killed and from those that survived characterisation or durability monitoring bioassays. A sample of each cohort of test mosquitoes could be stored for future analysis—for example, detailed characterisation of resistance mechanisms if results from one facility, or one-time point, varied from the others. This may be straightforward to compare changes in target site allele frequencies (e.g., kdr), but it may be more challenging for changes in metabolic gene expression, where defining a threshold of fold change is required, which would mean two populations were no longer comparable. Snap freezing at −80 °C would be ideal, so that relatively high yields of DNA and RNA can be analysed from stored samples, but even storage of dried individuals on silica would also be suitable for some further analyses.

If this approach were taken, differences in the resistance profile of strains used by different test facilities would still exist, and the strain or strains used may change in resistance phenotype during the study, but the robust characterisation and quality control should help to ensure that the key (known) parameters are similar enough, and that differences can be taken into account when interpreting data. This approach was selected for further development into the final recommended protocol. However, it was agreed that there would need to be a balance between the benefits of data robustness and the ability to reliably interpret results and compare across studies, and costs of additional workload required for extra bioassays and the ease of access to molecular characterisation. 

#### 3.1.4. Sample and Rear Wild Resistant Populations for Each Round of Testing and Save Samples for Characterisation

Where test facilities do not have access to a well-characterised resistant strain, or where issues, such as colony collapse or loss of resistance, result in non-availability of a suitable strain, a pragmatic alternative approach, sometimes employed, is to collect and rear wild resistant field populations for bioefficacy testing. Since wild-caught mosquitoes are likely to demonstrate large variability in the level of resistance and general robustness between collections, a cohort could be stored from each testing point for molecular characterisation. If sufficient material was available then phenotypic resistance and measures of fitness, including wing length, could be measured in parallel. While this approach still requires the team to have the capacity to maintain and characterise strains, less long-term commitment of resources may be needed, compared to holding strains over the whole course of the study. In some settings it may be very challenging to establish stable resistant colonies and using material maintained in the insectary for a generation or two to complete a study might be more practical. The biosafety concerns of transporting resistant mosquitoes between facilities can be avoided using local strains.

However, there would be a concern, especially when using F_1s_, that testing is a mix of different species; this could complicate interpretation of results, power calculations, and assay replicate requirements. The storage of samples for later analysis would also help with this element of characterising the testing cohort of mosquitoes. If a colony could be established for later testing points in the study the strain could then be screened regularly and become more well-characterised. 

For some purposes, using field-collected, or recently established, colonies of mosquitoes may be desirable. For example, it may be more predictive of field performance of an ITN than using established laboratory strains, since mosquito populations at different geographical sites may differ in their susceptibility to a given product [51], owing to the different resistance mechanisms they express, and potential for variability in levels of resistance across seasons [47]. Additional information would also be gained about the predicted ongoing efficacy of the nets locally by using locally-collected mosquitoes for durability monitoring, which possess field-relevant mixtures of resistance phenotypes. This may be important for NMCPs when making ITN procurement decisions, though this may not be the case if recently caught wild mosquitoes are being mixed in culture with previously colonised wild mosquitoes. However, bioefficacy testing for ITN durability monitoring requires capacity to detect a change over time, so reproducibility of results and consistent longitudinal use of a well characterised strain is critical. If tunnel tests are required for testing of a dual-AI net (e.g., Interceptor G2) wild collected mosquitoes are unlikely to be suitable, due to low levels of attraction to guinea pigs, which often results in low levels of blood-feeding success in untreated control tunnels. For this reason, the group saw this as a backup option rather than the primary approach for using resistant mosquitoes as part of durability monitoring or similar study. 

#### 3.1.5. Conduct All Testing in a Few Chosen Test Facilities

Depending on the study design and available resources, it may be possible to standardise all bioefficacy testing by sending all sample ITNs to a single facility or to a small number of test facilities. In this way, the number of mosquito strains used across the study would be minimised, reducing variability between data sets. Other potential sources of variability are also controlled for, such as operator differences or the effect of different testing conditions. Comparing data between time points in a study would be easier than compiling data from multiple test facilities.

On the other hand, the need to test a large number of samples in a single test facility might cause a delay in processing the collected net samples, with the associated risks of changes to the resistance level of the mosquito strain between the start and the end of testing. Although net samples can be stored in refrigeration, there is also a risk of degradation during storage. This approach provides no control for the mosquito population changing between time points. Outsourcing testing to a single or small number of testing centres is unlikely to fit within country-specific National Malaria Control Program (NMCP) capacity development objectives. It may present challenges to the funders of durability monitoring studies. This was not a preferred approach.

#### 3.1.6. Send All Samples to Several Labs for Repeat Testing in a Multi-Centre Study

Testing net samples in several laboratories could avoid the need to characterise mosquito strains in detail by testing the same samples against a different strain in each facility and evaluating result consensus by compiling the data, and assessing variability in results. Since the AIs may be unevenly distributed across a single ITN [52], giving different results from different samples of the same net, pieces should be cut along the same band to distribute to multiple facilities for parallel testing. Wherever possible, this testing would be done blinded.

This approach multiplies up the testing workload by the number of facilities. To reduce the additional workload, a study could circulate a sub-sample of net pieces to additional test facilities for confirmatory testing of the results obtained by the primary test facility, with careful consideration given to how to manage a situation where results did not match between facilities. Transporting ITN samples, particularly between countries, can be challenging. There is a risk of further degradation of samples, due to delay and during transport between facilities, and a requirement for each facility to colonise and maintain a resistant colony of mosquitoes. From a quality control point of view, it is good practice for a study to repeat testing on at least a subset of samples at different test facilities. It could be done intermittently as an additional level of quality control. However, from a logistics, and particularly from a cost, point of view, this would not be a feasible approach to standardisation for all studies.

#### 3.1.7. Measure the Added Effect of a Dual-AI ITN Relative to a Pyrethroid-Only Net

Although it would always be beneficial to understand the nature of the strain of resistant mosquito being used for testing, an additional or alternative approach to measuring the bioefficacy of the non-pyrethroid AI is to simply expose them to both a pyrethroid-only net and the dual-AI ITN under evaluation, and use the difference in mortality between the two as the endpoint. This approach would allow the comparison of the additional mortality induced by the second AI between time points to be used as a measure of continued bioefficacy. Where the endpoint caused by the second AI is different to the mortality caused by the pyrethroid, for example in the case of PPF, which causes sterilisation, no correction is needed and the level of sterilisation is the measure of the bioefficacy of the second AI. Evaluation of this effect is only possible by using a highly pyrethroid-resistant strain so that sufficient mosquitoes survive exposure to the dual-AI ITN and can be scored for fertility. This is conceptually a simple and attractive approach, removing pyrethroid content as a variable and controlling for variability between strains or changes in a strain over time, at least in terms of the pyrethroid resistance phenotype. However, this approach assumes that tolerance of a strain to the second AI does not change over time, so that even if the strain changes in its pyrethroid resistance, its response to the second AI remains constant. It also assumes that there is no cross-resistance, i.e., that the mechanisms conferring resistance to pyrethroids do not also confer resistance to the second AI. Subsequently, if susceptibility to one AI changes over time susceptibility to the second AI remains unaffected. This may be true for some new insecticides, but there is evidence of cross-resistance mediated by cytochrome P450 enzymes [53,54], including between pyrethroids and pyriproxyfen [55], so it cannot be assumed. 

If there is an interaction between the pyrethroid and the second AI in the formulated dual-AI ITN, then it may not be possible to make a straightforward comparison; the two AIs may not act independently, making a direct comparison between mortality on the pyrethroid-only versus the dual-AI net samples problematic. If it was possible to obtain comparable ITNs treated with each AI alone to compare bioefficacy of each with bioefficacy of the dual-AI ITN then a direct comparison could be made, and investigation of cross-resistance would be facilitated. On the other hand, the change in bioefficacy over time is relevant to a durability monitoring study. If the mortality caused by the pyrethroid-only net is sufficiently low it should still be valid to compare the additional mortality caused by the dual-AI ITN sample between time points. 

Pyrethroid content is only removed as a variable if the pyrethroid-only net is equivalent to the pyrethroid content on the dual-AI ITN, in terms of the identity and concentration of the pyrethroid, as well as factors that might affect bioavailability, such as ITN formulation and impregnation method. For example, incorporated and coated nets may have different surface concentrations of AI and consequent bioavailability even where the total insecticide content is the same. This comparison becomes complicated for combination nets, such as the PermaNet 3.0, where the pyrethroid content is different on the roof and on the side panels. The selected pyrethroid-only net should be as close as possible in all characteristics to the ITN under evaluation, particularly for insecticide dose and bleed rate (where known). For some dual-AI ITNs no suitable pyrethroid-only net is available. A specifically matched pyrethroid-only net would likely rely on manufacturers producing small batches specifically for the purpose. This is not realistic, without incentive such as making it a requirement as part of the WHO Vector Control Product Prequalification (PQ) process, for example, and so the closest matching net would have to be used. The positive control should be kept consistent between time points; it may not be essential to be consistent between facilities if the relative change in additional mortality from the second AI over time is measured. A definition of ‘brand new’ or positive control net would be needed, along with guidance on storage conditions, especially for newer brands of nets, a method for washing and washing interval for the insecticide’s regeneration.

Validation of the method against different second AIs using a range of resistant strains would be needed to have confidence in this approach, including the development of guidelines for the interpretation of results, establishing the threshold of killing when comparing the two nets, including the target minimum mortality among the resistant strain when exposed to the pyrethroid-only net, and gaining an understanding of the level of variability inherent in the assay. Additional controls could include exposing a susceptible strain alongside the resistant strain, including an unused and unwashed dual-AI ITN, or a net sample which only contains the second AI. However, this would likely have to be produced specifically for the strain characterisation by the ITN manufacturers, and again this is unrealistic without incentive. 

It was agreed that this approach does not give sufficient standardisation for the durability monitoring studies under consideration. It might be enough for other purposes, such as screening field populations known to be resistant to pyrethroids to inform deployment decisions, but the variation inherent in these tests would likely lead to such wide confidence intervals in the data that it would not be sufficient for providing evidence to the WHO PQ Unit for vector control products assessment (PQT/VCP) of continued bioefficacy as part of durability evaluation in a product dossier. However, the consensus was that including a pyrethroid-only net in durability monitoring bioassays as a control would be good practice, if suitable net samples are available. The specific characteristics of the control net (brand, batch number, age, polymer, insecticide type and concentration, coated or incorporated, storage conditions) should be reported alongside the assay results. A pyrethroid-only control is not equivalent to the pyrethroid content or presentation in the dual-AI ITN. It could still be used as a proxy indicator to help calibrate and interpret test results, rather than an exact one measure to infer additional mortality induced by the second AI directly. The additional control of a brand-new dual-AI net would also be a way to control for the variability of the resistant strain over time, though with some of the same practical caveats as above. More generally, comparison between the bioefficacy of a pyrethroid-only ITN and a dual-AI ITN will help to inform procurement decisions.

#### 3.1.8. Perform Bioassays of Nets from Multiple Time Points Side by Side at the End of the Study

To control for variability in the resistant mosquitoes used for testing over time or between test facilities, all nets sampled during the study could be stored and then tested in a short period at the end of the study. The major disadvantage of this approach is that information about the expected performance of the nets would not be gathered in real-time. Since durability monitoring is currently the main means of identifying quality issues with nets, this would have significant operational impact. There would also be the challenge of performing a large number of bioassays in a short period, rather than a smaller number at each time point, and the risk of a catastrophic event leading to loss of net samples from the whole study with no durability data being collected at all. Practical issues worthy of consideration are the potential for nets to degrade further during storage and the need for substantial storage space under specific controlled conditions. This approach to standardisation was agreed not to be suitable as a standalone standardisation measure.

A compromise would be to store a subsample of nets at each time point, after they have been collected back and used for bioassays, and repeat testing on this subsample at the end of the study, where resources allow. This has the advantage of confirming the results of bioassays conducted during the survey in side-by-side testing with minimal variation in the mosquitoes used, and could also be used to try to understand any unusual results observed during the study, supposing that data collected during the study were felt to be robust enough. In that case, a robust enough decision could be made to scale back this final testing or not continue at all, but the samples would be available as a backup. Additional standardisation measures would need to be taken during the initial bioassay testing performed during the study. Still, the group thought this could be a valuable addition to other characterisation or standardisation measures for WHO PQT/VCP studies, and monitoring of durability of nets in operational deployments. 

There is the opportunity to build this into existing durability monitoring protocols used by PMI-supported studies and others [56], where nets are removed from use, typically within six months and annually for three years, for durability monitoring and replaced with new nets. Currently, these replacement nets are excluded from any further monitoring, but by the end of the study would represent nets of ages corresponding to each time point of the study and could be collected at the end for a final confirmatory round of bioassays. Important caveats of this approach include: there would still be a large amount of testing to be done at the end of the study, a more significant number of replacement nets would need to be distributed to account for attrition and leave a large enough sample for the final testing, and careful record-keeping would be required as different batch numbers may be distributed at other time points adding a layer of complexity. Nevertheless, the additional quality control of data could be used to justify the additional logistics and expense of this approach.

Iinformation would need to be generated on the likely variability between original and replicate testing inherent in the bioassay, so that the results of the repeat testing could be interpreted judiciously. Consideration should be given to how to report results of this repeat testing, particularly if initial monitoring data have been distributed or published already; protocols published ahead of the trial could make it clear that this repeat testing is part of the study design and careful interpretation and reporting of results which do not completely align will be required.

#### 3.1.9. Use a Model System Other Than a Conventional Bioassay Using Mosquitoes of the Target Species

Conventionally, the durability of an ITN is tested using defined measurements of physical integrity, insecticide content and bioefficacy. For bioefficacy, cone bioassays, where the target mosquito species are exposed to a net sample, and the mortality is scored, is the accepted measure of field-collected ITNs over time [9]. Since the purpose of durability monitoring is to detect any change in bioefficacy of the net sample over time (i.e., ITN age), in a system that otherwise gives consistent results, the testing does not have to be against the vector species of interest. For bioefficacy testing in general, it is unnecessary to use the species against which a product will be targeted, as long as their relative sensitivities in a bioassay are understood. Aedes mosquitoes, particularly *Ae. aegypti*, can be reared in large numbers [57,58,59,60], with the added benefit of eggs resistant to desiccation, and can be stockpiled until sufficient eggs have been produced for a round of testing. It may even be possible to use a model organism, such as *Drosophila melanogaster*, to replace mosquitoes altogether, which has less of a containment risk and is easier to maintain, possibly expanding the number of test facilities able to perform durability monitoring. Validation would be needed to show that the chosen bioassay was appropriate for another species and that the species was sensitive to a change in AI concentrations across the relevant range. Even then, there may be reluctance to rely on results from a non-target species to test the efficacy of a product primarily aimed at anophelines.

New technologies are emerging which might offer a valid alternative to conventional bioassays or mosquito strains established from field-collected material. Transgenic strains of *Anopheles gambiae* over-expressing specific P450 enzymes, known to be important in conferring pyrethroid resistance, can be used to detect and characterise cross-resistance between insecticide classes [61]. A strain could potentially be produced that over-expressed the enzymes known to cause resistance to the second insecticide in a dual-AI ITN, expressing a very tightly defined resistance mechanism in a known genetic background. 

Measures of bioefficacy suffer from high variability due to inherent bioassay variation and biological variation between mosquito populations. Chemical analysis of the total insecticide content of a net sample, for example, by HPLC, may be more reproducible but is not sufficient as a measure of bioefficacy, since it is the availability of biologically active insecticide on the surface of a net that determines its efficacy against mosquitoes [39]. However, suppose it was possible to sample and quantify the amount of bioavailable insecticide on a net surface. In that case, this might be quicker than performing bioassays and an equally informative measure of bioefficacy. It would need to be correlated with the results of bioassays to be validated as a replacement method. 

Novel techniques and approaches warrant further investigation, especially as ITNs continue to evolve. However, a method to monitor the residual bioefficacy of dual-AI ITNs is needed urgently, precluding much analysis of available options or the development of new systems.

### 3.2. The Final Protocol: Characterisation of the Resistant Strain in Parallel with Bioassays

**Figure 1:**
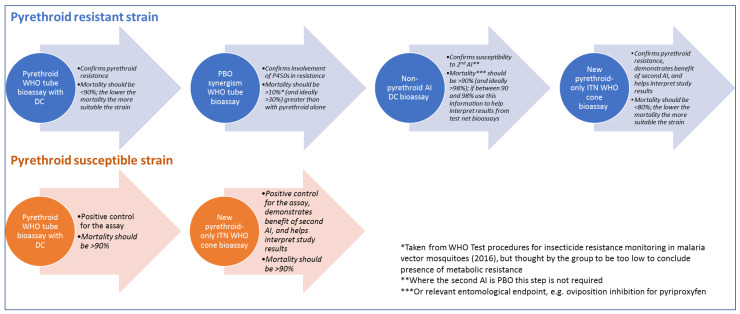
Overview of protocol for characterisation of a pyrethroid resistant strain for use in testing the bioefficacy of a dual-AI ITN, developed by consensus of a group of key stakeholders. Where delayed mortality (scored after more than 24 h) is the endpoint of interest for the second AI, mortality should be scored at this later time point for all elements of the characterisation; mortality may also be scored at 24 h.

The protocol for characterising the resistant mosquitoes used in bioefficacy testing with dual-AI ITNs is outlined in Figure 1, and a detailed standard operating procedure (SOP) is provided as Supplementary Information. The group agreed on this approach following several rounds of discussion on the merits of each of the proposed strategies and refinement of this preferred approach. The primary concern of the group was durability monitoring studies with dual-AI ITNs, but the protocol could be adapted to new types of ITNs as they are developed, to other product types, such as indoor residual spray (IRS) formulations, or attractive toxic sugar baits (ATSBs), and for other kinds of studies requiring resistant mosquito strains. 

Since resistance changes over time, in both wild mosquito populations and laboratory strains, even when consistent selection pressure is applied, the only way to be confident in the resistance phenotype at the time of testing is to characterise the resistant strain simultaneously with bioassaying of the ITN samples. Depending on the study design, describing each cohort of mosquitoes used for bioassays on net samples could be laborious. Instead, a resistant strain could be characterised at the start and end of a study, for example, all net samples collected in a given year or from a given district. The following elements were considered to be key to the characterisation:

- The proposed colony of mosquitoes would be exposed to a discriminating concentration (DC) of the same pyrethroid as is present in the dual-AI ITN in a WHO tube assay to confirm their resistance phenotype. If the mortality was above 90%, the WHO definition of confirmed resistance [42], an alternative strain should be identified to complete the testing. Below this threshold, a strain with as low mortality as possible should be used to maximise the data generated to test the efficacy of the second AI.
- The proposed strain characterisation includes a PBO synergism assay to confirm susceptibility. Where a DC method has not been established or recommended by the WHO, and baseline susceptibility has not been demonstrated, some suitable method of exposure to the second AI should be included in the characterisation, and data monitored for changes in susceptibility over time. Mortality should be above 90% and ideally above 98%; values between 90 and 98% can be used to interpret the results of the main study.
- A PBO synergism assay is included in the proposed strain characterisation to confirm that metabolic mechanisms, most notably those associated with cytochrome P450 enzymes, are involved in the pyrethroid resistance of the strain used for bioassays. The group agreed that further investigation is warranted to determine what increase in mortality with pre-exposure to PBO indicates significant synergism, but suggested that the current WHO test procedures threshold of 10% is far too low to account for realistic variability in estimates [42]. Provisional analysis suggests that a mortality increase of 30% is required to provide meaningful evidence for impact.
- Standard untreated nets or solvent-only controls should be included in the assays used to characterise a resistant strain, with some additional controls. These represent a balance between gaining confidence in the assay and additional information against keeping the additional testing for strain characterisation to a manageable scale.
  - A positive control brand-new pyrethroid-only net, containing the same pyrethroid content as the dual-AI ITN, should be included, for several reasons. Firstly, it provides an additional measure to ensure the strain has sufficient pyrethroid resistance, and will help interpret the results from the sample ITN bioassays. Pyrethroid content is controlled for as a standard variable used to calibrate results between time points in the test net samples. Finally, exposing mosquitoes to a brand new pyrethroid-only net alongside the dual-AI test nets would demonstrate the added benefit of the second AI. Where multiple brands of such nets are available, the net most similar to the test net without the addition of the second AI should be selected, where possible of the same material and with the correct AI applied similarly (incorporated/impregnated) at the target concentration and release (bleed) rate. The brand should be consistent across the study.
  - Including a susceptible reference strain alongside the resistant strain acts as a control that the bioassay is functioning as expected, confirming the potency of WHO filter papers and pyrethroid-only net samples. It also serves as a benchmark to demonstrate the additional benefit of the second AI over that of the pyrethroid. Minimum mortality in the susceptible strain exposed to the DC of the pyrethroid in the dual-AI ITN and a brand new pyrethroid-only net should be 90%.
- An assessment of body size is included as a further quality control measure to help to interpret results with more confidence. Wing length is the recommended measure, but dry weight could be more practical. Size varies between species and rearing facilities, so it is not appropriate to set absolute thresholds, but collecting size data alongside the bioassay results is still valuable in helping to interpret bioassay results. For example, an unusually small cohort may explain anomalously high mortality in a bioassay [27].

Where both AIs induce mortality, the endpoint measures for the two AIs should be the same. If the outcome for the second AI is delayed mortality, then mortality caused by the pyrethroid should be measured for the same period. For example, in products containing chlorfenapyr, mortality is typically measured to 72 h [30,42], and so mortality should also be measured to 72 h for the pyrethroid treatments in the strain characterisation. This would control for additional delayed mortality in the resistant strain caused by the pyrethroid, which has been measured in some, but not all, strains tested [62,63,64], which could mean that for the purposes of the study the strain was not sufficiently resistant. Scoring knock-down and 24 h mortality for the pyrethroid exposure as well might be useful for comparison with historical data. If the second AI induces a different endpoint, for example sterility, and the study is aiming to measure the effects of each AI separately, then it would be necessary to include investigation of pyrethroid exposure on that endpoint.

A number of additional measures were recommended by the group as general good practice and to further characterise and standardise the resistant mosquitoes used for bioefficacy testing:

- It is desirable to use a strain with the same phenotype throughout a study. Efforts should be made to minimise heterogeneity of strain phenotype through time by standardising insectary rearing procedures, since insecticide susceptibility is affected by the size and general fitness of the cohort of insects used for testing. Standardisation of rearing conditions is especially important for strains used to test products such as chlorfenapyr, where metabolic activity is important in activating the insecticide and affected by rearing conditions and conditions during testing [25,65]. Although it may be unrealistic to ask for a single rearing SOP to be used between facilities, most facilities use some form of SOP to achieve standardised rearing and perform quality control (QC) measures, particularly those with GLP accreditation [66,67]. There are guidance documents available [48]. Some key considerations for maintaining the consistent quality of mosquitoes being reared for bioassays and steps taken to monitor quality are suggested in Table 2. Quality management systems help ensure that the data generated is reliable and reproducible and that it is possible to reconstruct a test in case there are any questions about data quality from manufacturers, regulatory authorities, etc.
- Strains are typically maintained under selective pressure and characterised routinely. These efforts could be enhanced by increasing the frequency of QC activities and including frequent selection and profiling with the pyrethroid of interest to the study. These data could be provided alongside the durability monitoring instead of parallel characterisation. Composite fitness indices can characterise mosquito populations used in experimental settings [68].
- If sufficient data for the colony exists, it is recommended to set upper and lower size thresholds, based on variability of size measured in the colony over a period of time, outside which testing would not proceed.
- The group recommended that a sample of mosquitoes be stored at the time of strain characterisation so that it could be characterised in more detail later if required to help explain an anomalous result, such as a drop in pyrethroid resistance or an increase in mortality from the second AI compared to a previous round of testing
- Inclusion of a brand-new dual-AI ITN of the same type as the test net samples as a control in the strain characterisation for durability monitoring studies would provide the following benefits (note that replicate pieces would be needed due to variation between and across an ITN):
  ◦ Control for longitudinal variability in strains
  ◦ Be a second measure of how much of the original bioefficacy has been lost over time in addition to the comparison between results obtained at the different time points
  ◦ Allow the additional mortality caused by the dual-AI ITN over that of the pyrethroid-only net to be calculated at each time point and compared longitudinally
  ◦ Control for any effects of declining content of the first AI over time. This is particularly important as the wash resistance of the pyrethroid and the second AI may be different, and so the additional benefit of the second AI may be lost before that of the pyrethroid
- Although a WHO tube assay is a standardised method to measure synergism, a cone test with a PBO net would provide a more realistic presentation of the PBO in combination with a pyrethroid because of simultaneous exposure. If a suitable PBO ITN is available as a comparator for the dual-AI being tested, for example DuraNet as a comparator for the DuraNet Plus or Olyset as a comparator for Olyset Plus, then cone tests with this ITN may be informative in more accurately evaluating the level of PBO synergism, and so metabolic resistance status of the resistant strain.
- SOPs will be required to collect, store, and transport net samples, both those tested and the control nets used for characterisation. The storage conditions and the maximum storage length are essential for incorporated nets. Typically, cut pieces are wrapped in foil and stored in a fridge. However, the development of these is outside the scope of this document.

It is strongly recommended that the results of strain characterisation be presented alongside study data to aid the interpretation of bioefficacy results. An example of how this might be done is shown in Table 3.

As an additional standardisation measure, the group proposes for durability monitoring bioassays that a sub-set of dual-AI ITN samples is retained from each study time point to repeat bioefficacy testing, and characterisation of the strain, with ITN samples from all time points in parallel at the end of the study. Suppose the nets are stored appropriately to minimise the degradation over time, in that case, this additional test allows for a direct comparison between samples to minimise the difference in the mosquito population and reconfirm the trend in mortality measured over time during the study. Since the bioassays were also performed during the study, a data set would still have been generated if storage conditions turned out unsuitable and samples were degraded, lost or damaged over time.

An alternative to the retention and repeat testing of nets at the end of the study may be possible and has some advantages as an additional standardisation step. At each time point during a durability monitoring study, a sample of nets is collected from the field for destructive sampling (i.e., bioassay) and replaced with new nets of the same brand to prevent the household from being left unprotected. At the end of the study, a sample of these replacement nets could be collected alongside the nets being sampled for the final time point, and bioassays performed on all nets in parallel. In this way, nets of all ages could be tested side by side for a more direct comparison, with the same characterised strain of mosquitoes [56]. This approach avoids the risk of degradation of nets collected at each timepoint and held until the end of the study for parallel repeat testing. A more complicated study design is required, and additional nets would have to be distributed to ensure sufficient nets remained at 36 months, since nets get discarded as they wear out. In carefully conducted research studies that employ unique labelling of individual nets, it should be possible if additional cost could be supported but not feasible for programmatic evaluations.

### 3.3. Considerations and Points of Discussion in Deciding on the Final Protocol

#### 3.3.1. Sample Size

When producing data to characterise a mosquito strain, the more mosquitoes tested, the more robust the result, achieved by increasing the number of replicate assays (cones, tubes or bottles). Given the inherent level of variability in the bioassays proposed, it would be desirable to recommend a minimum number of replicates on which a result should be based. The protocol proposed here uses the WHO test procedures for resistance monitoring [42] as a baseline measure of how many replicates are required for each assay, but as more data are produced using this protocol, more robust power calculations, or the application of modelling, can be used to refine the recommendation. However, if F_1_ mosquitoes are used for testing upward-adjustment of sample sizes might be considered because of greater inherent variability compared to (inbred) laboratory strains [40], and is essential if species mixtures are expected. 

#### 3.3.2. Controlled Conditions during Characterisation of Strains

As with bioefficacy testing generally, it is necessary to control the climatic conditions during the strain characterisation bioassays. At minimum, the temperature, relative humidity, and time of day should be recorded and closely monitored in case of electricity cuts or other fluctuations. A reporting checklist would be helpful to encourage accurate reporting, whether the SOP is followed thoroughly or whether deviations have occurred for whatever reason. This will aid downstream interpretation of the results, and if temperature or humidity variation is implicated in production of apparently aberrant results, repetition of tests that were conducted out of specified ranges is advisable. Additionally, depending on the nature of the second AI, the time of day the bioassays, both strain characterisation and durability, testing are conducted might be critical [26,70,71,72]; for example, in evaluating a dual-AI ITN containing chlorfenapyr [25]. Some key parameters to consider standardising when performing bioassays with mosquitoes are suggested in Table 2.

#### 3.3.3. The Approach Selected Must Be Applicable in Most or All Test Facilities

Proposed protocols must be practical, affordable, safe, and accessible in strain availability and facilities to maintain and characterise mosquitoes. The more criteria for suitable strains in place (multiple resistance mechanisms, resistance levels, characterisation methods), the more difficult it might be for test facilities to meet these criteria.

### 3.4. Deciding on Criteria for a Suitable Resistant Strain

The group’s discussion over what criteria to set for a resistant strain was an attempt to strike a balance between a desire to characterise the strain in the greatest possible detail, to allow the best interpretation of data and comparison between data sets, and the pragmatic considerations of how much additional resource burden could be borne by programmes evaluating the dual-AI ITNs. The final criteria agreed by the group is highlighted in the protocol overview in Figure 1.

There was an agreement to recommend using a single well characterised strain for all testing within a study to remove this as a possible source of variation. ITN efficacy testing may be important to test against resistant strains of all significant *Anopheles* vector species, but this is not critical for durability monitoring. Indeed, the species used for the bioassays need not be a target of the ITN at all. As long as it is validated for the assay, it was sensitive to changes in bioavailable AI and relevant concentrations of the second AI.

There was a preference to use a strain with resistance conferred by multiple mechanisms, to produce the most widely applicable results; however, to confirm the presence of multiple mechanisms is complex and beyond the capacity of many test facilities. Although resistance to the first AI, currently always a pyrethroid, in the dual-AI ITN is the relevant requirement of the mosquito strain, and sufficient susceptibility to the second AI is required, a broader resistance profile might be desirable. It is likely sufficient to demonstrate resistance to the specific pyrethroid in the product under evaluation. Still, there may be a benefit to knowing that multiple resistance mechanisms are acting and using a strain shown to be resistant to pyrethroids in general and other insecticide classes. The consensus was that more information may always be desirable, and would help explain variable results across time or between test facilities. Still, an understanding of the resistance mechanisms present is probably not necessary for the question at hand.

Overexpression of cytochrome P450s appears to be the mechanism most commonly implicated in metabolic resistance and cross-resistance [53,56]. So, upregulation of P450s would be a desirable minimum criterion in a resistant strain. This could be demonstrated by characterising expression levels of a panel of key enzymes in the resistant strain (as detailed in [19]), and the potential for cross-resistance with the second AI of interest could be predicted with the use of P450 screens [54]. To adequately describe a strain’s metabolic resistance risk, the most relevant molecular markers would need to be identified, along with the P450s most important in conferring resistance to the first AI, and then acceptability criteria based on fold-increase in expression relative to a susceptible strain would have to be established. This is challenging, however, and a strain showing a broad overexpression profile, including at least some known key enzymes (with proven insecticide metabolic capacity), may be more realistic. The interaction of P450s with the second AI would ideally be characterised as well. These analytical methods are specialised and relatively expensive, but regional reference laboratories may support programmes in analysing mosquito strains for this purpose. Given restricted resources, a programme could set out to molecularly-characterise the key strain, or strains, used for bioefficacy testing in durability monitoring at least at the start of the study. However, P450 expression levels are likely to change over time, particularly under selective pressure usually applied to laboratory-maintained resistant strains, so repeated analysis, perhaps of a reduced set of key markers or enzymes identified during initial characterisation, is desirable.

Given the costs associated with a more sophisticated analysis of resistance mechanisms, a pragmatic alternative is to demonstrate the involvement of metabolic resistance (primarily attributable to P450 enzyme activity) in the selected strain using a PBO synergism assay. Demonstrating that mortality is increased by PBO pre-exposure followed by a pyrethroid exposure relative to a pyrethroid alone may be sufficient to demonstrate the presence of P450-mediated resistance. This could be done with a WHO tube assay [42] or exposure to a locally relevant pyrethroid-PBO ITN, which would give useful efficacy data relevant to the local setting. Moving away from the standard protocol would make comparing facilities more challenging, though the standard synergism assay may not always be very informative [40]. Testing with other synergists might be informative, as might testing the effect of PBO pre-exposure followed by exposure to the second AI. Still, standard methods have not yet been established [41]. 

The most pragmatic way to determine that a strain is suitable for monitoring the durability of the second AI in a dual-AI ITN is to confirm its resistance to the first AI, and ensure that it meets the acceptable criteria of mortality in a standard bioassay. This can be done through WHO tube bioassays using 1×, 5× and 10× DCs and selecting strains that are, for example, at least moderately resistant (<90% mortality at 5× DC) according to WHO definitions [42]. The resistance level could be determined more precisely using dose-response experiments to calculate LC_50_ values and resistance ratios relative to a susceptible comparator strain. If a standard SOP was used, these results could be compared between test facilities. Criteria that a minimum fold-increase in resistance be met before a strain was used for durability monitoring could then be set, though this is a labour-intensive approach, particularly since the LC_50_ for a susceptible population would ideally be set using multiple susceptible strains in a multi-centre study, to overcome the noise that is inherent in this approach. There may not be a need for strict resistance criteria since durability monitoring simply needs to detect a change in bioefficacy over time. However, a sufficient proportion of the exposed mosquitoes must survive exposure to the first AI to allow detection of an effect of the second AI.

It is important to measure the susceptibility of the resistant strain to the second AI in the product under evaluation in the absence of the first AI as part of strain characterisation. Even where an insecticide has previously not been used for mosquito control, resistance may have emerged as a result of agricultural use [2]. There is also the potential of cross-resistance to an insecticide with a different mode of action in mosquitoes resistant to pyrethroids, possibly through more general mechanisms that increase metabolism or reduce penetration. For example, the same metabolic enzymes appear to target pyrethroids and pyriproxyfen [56,57,73]. Programmes measuring the efficacy of a new vector control product should monitor the target population for emerging resistance. Still, it is also desirable to show that the resistant strain used to test the durability of the second AI does not already have a level of cross-resistance to it and that such resistance does not develop during the study. For PBO products this can be established during characterisation of pyrethroid resistance, as described above, and where the WHO recommends a DC and suitable methodology, this can be built into the strain characterisation [42]. Where such a method is not available for the second AI, cross-resistance may be predicted through molecular analysis [54], but this would normally need to be the subject of substantial additional investigation.

It is possible that the methodology selected for the bioefficacy component of durability monitoring could affect the criteria for a suitable resistant strain. For example, the cone test and tunnel test are very different modes of exposure and environments in which mosquitoes encounter a net sample for different exposure times and a strain that is not killed by the pyrethroid in an ITN in a cone test may be killed in a tunnel test. Where a non-standard methodology is used to measure bioefficacy, it is recommended that data from the baseline bioassays with a selected resistant strain be reviewed along with the data from the strain characterisation exercise to confirm that the strain and standard bioassays are suitable for that specific study. 

### 3.5. Cost Implications of Adding Strain Characterisation to a Study

The development of this characterisation protocol seeks to outline an optimum method to characterise and standardise resistant strains for use in bioefficacy testing of dual-AI ITNs. These efforts are required to produce robust and reliable data, but additional funds will be needed to support the additional testing. Following the SOP detailed in Supplementary Information would add a workload consisting of six WHO tube assays with a pyrethroid, six WHO tube assays for the synergist experiment, six DC assays for the second AI and five cone tests with a brand new pyrethroid-only ITN with the resistant strain, plus two WHO tube assays with a pyrethroid and five cone tests with a brand new pyrethroid-only ITN with a susceptible reference strain, a total of 475 resistant and 75 susceptible mosquitoes. If multiple types of dual-AI ITNs were included in a study, six additional DC bioassays would need to be added for each non-pyrethroid AI in the study, plus additional new dual-AI ITN positive controls, if included. The same mosquitoes can be used for QC and samples stored for later analysis, but these steps will require time commitment and consumables. Wing length analysis requires access to a microscope and image software or graticule, and further molecular analysis of samples may be required.

For a single experiment strain characterisation would be a one-time cost. Still, for a study lasting up to a month, strain characterisation should be completed before the study and repeated at the end of the study. Characterisation should also be repeated for longer studies, to ensure that the resistant strain has not changed in resistance profile and is still suitable, within one month of finishing, and where possible repeated during the study, on every mosquito generation if possible, or as often as practical. If resources were available to include some elements of the characterisation alongside each bioassay session, it would characterise the strain and provide an internal control for any differences between time points arising from changes in rearing, testing conditions, operator differences etc.

This additional cost may be small and easily borne for small scale research or development activities by academic institutes or developers or manufacturers of insecticide-based vector control tools. ITN evaluation and procurement is, however, a very price-sensitive market. Adding additional testing to the durability monitoring protocol will add cost to already expensive trials of new ITNs [74]. Durability monitoring is largely a donor-funded activity that is already growing in scale due to more complicated bioefficacy testing methodologies for dual-AI ITNs than was required for pyrethroid-only ITNs. The benefits of these additional characterisation steps will need to be accepted by funders, including the potential costs incurred should poor quality durability monitoring results lead to poor decisions on ITN choice. Decisions to procure more expensive ITNs can be made with greater confidence if the durability monitoring data are more robust in demonstrating their residual bioefficacy. Additionally, the scale of additional testing may be relatively insignificant compared to the bioefficacy testing already included in a study. For example, one reported durability study of ITNs in Madagascar required 50,000 mosquitoes to test 400 net samples [6]. The proposed protocol has been divided into minimum essential and additional desirable steps based on available resources. An exercise to calculating the cost of the characterisation and in scoping the willingness of funders to support may help promote the adoption of this proposed protocol. It is also likely that test facilities will support the minimum essential strain characterisation from multiple funding sources as it is incorporated into their regular facility running costs.

## 4. Discussion

Resistance to insecticides used to control mosquito vectors of disease is widespread, strengthening and evolving in the face of selection pressure from a limited number of chemistries available for use in public health [75]. New generations of insecticide-treated nets (ITNs) are now available based on novel mode of action chemistries, and other novel insecticide-based tools are in development to address this challenge. Dual-AI ITNs, including those containing two insecticidal compounds and a single insecticide paired with a synergist, promise greater effectiveness against pyrethroid-resistant mosquitoes. It is intended that the partner AI will have an effective lifespan of three years to match that of the pyrethroids currently in use, so that the new ITNs will fit into the existing campaign framework and contribute to resistance management. The dual-AI ITNs do, however, present a challenge in measuring their bio-efficacy in a laboratory setting, which is required to monitor their effective life through durability monitoring studies. Existing methods designed for ITNs containing only pyrethroids may not be suitable for those containing different modes of action insecticides or synergists. There is a need to test them against pyrethroid-resistant strains to quantify the entomological impact of the second AI. 

Bioassays are an important proxy test for the surface availability of AI, and for demonstrating the efficacy of ITNs in killing mosquitoes under standardised conditions. As our understanding of resistance mechanisms increases, so does the complexity in determining relative contributions and how they affect the bio-efficacy of different chemistries and formulated products. These dynamics may alter with changing parameters, such as surface concentrations of AI declining over the lifespan of an ITN. There is, thus, the potential for the introduction of great variability into the results of bioassays designed for pyrethroid susceptible subjects, when considering the specific characteristics of the pyrethroid-resistant strain used, as well as methodological issues related to the mode of action of the ITN. To help minimise the noise in bioassay results due to these various factors, it is imperative that we clearly define or describe material inputs into these studies. A key aspect of this is to standardise or characterise the mosquito strains being used in these assays as far as possible, to provide interpretable data for analysis and to allow the comparison of results over time, between products and between testing centres. In an operational setting there is inevitably a need to balance improved characterisation or standardisation of inputs with the availability of suitable controls and logistical and financial constraints.

This paper describes a collaborative effort by researchers and implementers interested in insecticide resistance and evaluation of ITNs to agree on an approach to characterise mosquito strains to evaluate dual-AI ITNs and a set of specific criteria for the phenotype a suitable strain should have. Such an approach to method development, while somewhat time-consuming, does allow those implementing these activities to agree on a standardised method. This approach could be applied to other sources of potential variation in vector control efficacy and/or durability studies. For example, current guidelines for monitoring durability of ITNs were developed for pyrethroid-based nets but have been adapted and updated for dual-AI ITNs through a similar consensus approach by Innovation to Impact (I2I) [30]. 

Care was taken in designing a methodology for strain characterisation to ensure a comprehensive, robust approach, feasible in the context of the level of effort needed from those facilities carrying out this work. The standard operating procedure (SOP) decided upon and presented here (Supplementary Information) identifies some key parameters for characterisation, presents criteria for a suitable strain, and provides guidance on the rearing and quality control of the mosquitoes used in testing. Components are separated into those which are critical and those which are desirable and should be included where resources and logistics allow. Although these recommendations may have cost and time implications, these are balanced by promise of greater interpretability of the data produced in notoriously difficult studies to analyse and compare. The SOP will be made freely available through Innovation to Impact (I2I), to be trialled. Future studies with dual-AI ITNs, such as durability monitoring activities currently underway [76,77], allow its suitability to be reviewed and the methodology to be refined based on the experience of operators.

The consensus recommendation of the group of experts was to use a laboratory strain of mosquitoes for durability monitoring of dual-AI ITNs, to allow controlled rearing, quality control and characterisation to maintain and monitor the consistency of material over time. Regular and thorough characterisation of laboratory strains used for longitudinal bioefficacy testing is critical to ensure data validity and reliable interpretation of findings. There was some discussion favouring using locally relevant mosquitoes, and a desire to determine the operational significance of strain characterisation of bioefficacy data generated in the laboratory. Although the goal of durability monitoring is separate from efficacy testing, if the latter is a key question, there may be a preference for testing nets against local strains or against multiple recently-colonised resistant strains, which may express different, but locally-relevant, resistance mechanisms and give additional information about how the nets perform in situ. However, durability monitoring aims to determine that over multiple geographical locations ITNs continue to remain physically and biologically active for the duration of their expected lives. This is particularly difficult for dual-AI ITNs that must be tested against resistant strains to ensure the non-pyrethroid component is still biologically active. For longitudinal experiments, such as durability monitoring or RCT trials, it is appropriate to use a well-characterised and consistent strain of mosquitoes. When a dual-AI ITN is being tested, it is critical to know the pyrethroid resistance phenotype of the mosquitoes being used. Positive control nets are a useful benchmark for interpreting changes in relative bioefficacy of dual-AI ITNs through time against a background of slight fluctuations in resistance phenotype of laboratory test strains.

The scope of this consultative exercise was the efficacy testing of the second AI in a dual-AI ITN combining a pyrethroid with a second insecticide or synergist. The 2022 Product Review Report from the WHO PQT/VCP team on insecticide treated nets formulated with a pyrethroid and either PBO or a second AI [78] recommended the development of ‘improved guidance regarding the selection of mosquito strains to be used in bioassay and efficacy testing’ including characterisation of resistance. The proposed strain characterisation approach addresses this need, and could be used in any situation where a pyrethroid-resistant strain is used in research. The general approach of characterising the biological material used in research and reporting results of the characterisation alongside the experimental data to aid interpretation is recommended as good practice. For example the WHO PQT/VCP Product Review Report [78] recommends the characterisation of the local vector population at the sites of experimental hut trials. This method establishes a solid framework that could be used with minor modifications to adapt to ITNs with unique AIs as they develop and become available. For example, specific additional or alternative considerations may apply when characterising a strain used to test ITNs containing two non-pyrethroid AIs. In this case, there will likely not be populations of mosquitoes available that are resistant to either AI. An alternative method would be needed to separate and measure the activity of each AI; for example, based on their differential speed of action. However, the requirements for maximising consistency and characterising the mosquito strain used to test the durability of these nets would be the same. The approach could also be readily adapted for characterisation of strains for evaluation of dual-AI products beyond nets, such as IRS formulations.

The development of this consensus methodology is part of a wider effort spearheaded by I2I to identify and address sources of variability in entomological data related to vector control product evaluation. To produce robust data, consistent across time and between operators, and to interpret results in a meaningful way, it is important to standardise or characterise material inputs into studies. The proposed method for characterising pyrethroid-resistant mosquitoes is the first of what is hoped to be a collection of supporting SOPs generated by, and made available to, the vector control community to help improve the generation and interpretation of entomological data for decision making.

## 5. Conclusions

To meaningfully interpret the results from bioassays and compare results between experiments it is important to maintain maximum possible consistency by standardising or characterising experimental conditions and inputs. When testing vector control tools, the target insect is a critical input. This work developed a method to characterise the resistance phenotype of pyrethroid-resistant mosquitoes used for bioefficacy testing of dual-AI ITNs. Adoption of this pragmatic yet informative approach will help in the interpretation of data from durability monitoring studies of these new net types. The approach can be adapted to characterise mosquitoes in other research involving biological materials where characterisation will help to generate consistent data which is more readily interpreted and compared, or where insecticide-treated materials are being used experimentally.

## Figures and Tables

**Table 1:** Possible approaches to achieve sufficient standardisation or characterisation of pyrethroid-resistant mosquitoes used for bioefficacy bioassays of dual-AI ITNs to compile, compare, and interpret results across studies. All approaches were proposed and considered by members of the stakeholder group and a consensus opinion reached as to their suitability and practicability; these are listed from most to least preferred or feasible approach.

Approach	Advantages	Disadvantages	Consensus Opinion
Characterisation of the resistant strain in parallel to the durability monitoring testing (see Section 3.1.3 below)	Simple methods available to characterise phenotypic resistance to most AIs Allows clear criteria to be set for a suitable strain Generates useful information for interpreting testing results	Standardised rearing and quality control measures also needed Strains may still differ between test facilities, though within acceptable thresholds	Robust strain characterisation and quality control of mosquitoes ensure that mosquitoes are similar enough to compare data between test facilities and across time, and help to interpret inconsistencies. **Approach selected for further development into the final protocol.**
Sample and rear wild resistant populations for each round of testing and save samples for characterisation (3.1.4)	Does not rely on all test facilities having suitable strains May require less resources than maintaining strains long term May be more predictive of local product efficacy; considers field-relevant resistance mechanisms	Wild-caught mosquitoes highly variable, some characterisation recommended Testing cohort might be a mix of species Wild-caught mosquitoes less responsive in tunnel tests	Consistency of mosquito strain is important to detect any change in response to an ITN over time, not achieved by this approach. **Back-up solution to colony collapse or loss of resistance, rather than the primary approach to characterisation.**
Send all samples to several labs for repeat testing in a multi-centre study (3.1.6)	No need to characterise mosquitoes Consensus data is generated which may increase confidence in the result	Higher testing workload, and each centre must maintain a resistant mosquito strain Risk of ITN sample degradation during transport Need for transport of samples between test sites	It is recommended as an additional step for quality control in a study to repeat testing on a sub-set of ITN samples at an additional site or sites. But costly and logistically challenging. **Not recommended as the primary approach to standardisation.**
Perform bioassays of nets from multiple time points side by side at the end of the study (3.1.8)	Controls for variability in resistant mosquitoes over time and between test facilities	Data not available until the end of the study, and real-time data are used to identify quality issues with ITNs Large testing volumes Risk of loss or degradation of ITN samples before testing	Repeat testing of a subsample of ITNs at the end of the study is recommended as a supplementary standardisation approach. Could test replacement ITNs to allow all ages to be tested in parallel. **Not suitable as a standalone standardisation measure.**
Each test facility uses its own characterised resistant strain with a single standardised protocol (3.1.2)	Conceptually simple additional step in method validation Additional information about local mosquito strains Capacity building from colonising and characterising strains	Validation required against multiple strains (≥3), likely as a multi-centre study Pyrethroid resistance varies between strains, complicating interpretation	Comparability between data collected at different facilities with different strains is a major issue. Unlikely that testing method would be robust enough to give consistent results between sites and across time points, regardless of the strain. **Not the preferred approach.**
Conduct all testing in a few chosen centres (3.1.5)	Reduces the mosquito strains used in the study Controls for other sources of variability between test facilities	Delays caused by large testing volumes Risk of ITN sample degradation during transport and storage Little opportunity for capacity development	Unlikely to be an attractive solution for in country programmes of funders of durability monitoring studies. **Not the preferred approach.**
Measure the added effect of a dual-AI ITN relative to a pyrethroid-only net (3.1.7)	Conceptually simple Removes pyrethroid content as a variable Controls for variability between strains or within a strain over time	Vulnerable to changes in susceptibility to the second AI over time, and interaction between the two AIs in the formulation Assumes lack of cross-resistance Relies on the existence of suitable comparator pyrethroid-only ITN	Not sufficient as a standalone standardisation measure for durability monitoring. Including a pyrethroid-only net as a control is recommended, particularly if a suitable comparator is available. **Not the preferred approach.**
Distribute the same well-characterised resistant and susceptible strains to all test facilities (3.1.1)	Straightforward standardisation Only one strain needs to be characterised Strain differences removed as a variable	Validation needed for each dual-AI ITN Colony resistance phenotypes and mechanisms may diverge after distribution Biohazard risk in transferring resistant strains	Transporting insecticide resistant strains between sites within known or potentially habitable range of species is not acceptable due to biohazard risk. **Not feasible.**
Use a model system other than a conventional bioassay using mosquitoes of the target species (3.1.9)	Could use a more amenable species, or very targeted or tailored approach Could replace bioassays with a quicker, more robust method	Equivalency would need to be established, and acceptability might be an issue	Further investigation to identify or develop new methods recommended for future use. **Suitable method not yet available.**

**Table 2:** Some key considerations for maintaining consistent quality of mosquitoes being reared for bioassays and conditions during bioassays, and steps that can be taken to monitor quality of mosquitoes.

Parameters to Standardise When Rearing Mosquitoes	Suggested Quality Control Processes in Mosquito Rearing	Parameters to Standardise When Performing Bioassays
Temperature Relative humidity (RH) Controlled light/dark cycle 1 h ‘dawn’ and ‘dusk’ Larval density and feeding regime Adult density in cages Non-limiting access to a sugar solution	Follow detailed rearing SOP Routine monitoring of some fitness indicator/s ^1^ to follow colony health and rearing quality Regular selection with at least one insecticide Periodic profiling of resistance phenotype Maintain staff training records on SOPs covering rearing and quality control Equipment maintenance and calibration Keep a record of deviations from SOP	Temperature Relative humidity (RH) Time of day ^2^ Feeding status (sugar, water, blood) Age of mosquito Measure a fitness indicator in testing cohort Maintain staff training records on SOPs covering testing, data handling, archiving etc.

^1^ In decreasing order of preference): composite fitness indices, wing morphometrics [69], wing length, dry weight, wet weight; ^2^ Mosquitoes may be reared on an adjusted light cycle to accommodate testing at a specific point in their circadian rhythm within working hours.

**Table 3:** Characteristics of a pyrethroid resistant and susceptible mosquito strain used for bioefficacy monitoring of dual-AI nets (an example is a strain used to monitor Interceptor G2, chlorfenapyr + alpha-cypermethrin ITN). Recommended format for presenting the results of strain characterisation should be provided alongside bioefficacy testing with dual-AI ITNs.

**Pyrethroid Resistant Mosquito Strain:** Tiassalé 13 **Species:** *An. gambiae s.l.*	
% Mortality (24 h) in WHO tube bioassay with alpha cypermethrin (0.03%)	54% (*n* = 94)
% Mortality (24 h) in WHO tube bioassay with alpha cypermethrin (0.03%) + PBO (4%)	92% (*n* = 96)
% Mortality (72 h) in WHO bottle bioassay with chlorfenapyr (100 ug/bottle)	100% (*n* = 97)
% Mortality (24 h) in cone test with new pyrethroid-only ITN (Interceptor)	70% (*n* = 106)
**Pyrethroid susceptible mosquito strain:** Kisumu **Species:** *An. gambiae s.l.*	
% Mortality (24 h) in WHO tube bioassay with alpha cypermethrin (0.03%)	100% (*n* = 90)
% Mortality (24 h) in cone test with new pyrethroid-only ITN (Interceptor)	95% (*n* = 103)
NB. All results Abbot’s corrected where control mortality was between 5 and 20%	

## Data Availability

Not applicable.

## Supplementary Materials

The following are available online at https://www.mdpi.com/article/10.3390/insects13050434/s1: I2I-SOP-004: Strain characterisation of resistant mosquitoes for monitoring bioefficacy in ITNs treated with two active ingredients (Dual-AI ITNs) [79,80,81].

## Disclaimer

The findings and conclusions in this report are those of the author(s) and do not necessarily represent the official position of the U.S. Centers for Disease Control and Prevention or the Department of Health and Human Services, or the U.S. Agency for International Development. Use of trade names is for identification only and does not imply endorsement by the Centers for Disease Control and Prevention, the Public Health Service, or the U.S. Department of Health and Human Services, or the U.S. Agency for International Development.
