# Supplementary material for: Strain Characterisation for Measuring Bioefficacy of ITNs Treated with Two Active Ingredients (Dual-AI ITNs): Developing a Robust Protocol by Building Consensus
Source: Insects. 2022 May 6;13(5):434. doi: 10.3390/insects13050434 (PMC9144861; doi:10.3390/insects13050434)
Supplement: Supplementary file 1 [file insects-13-00434-s001.zip › insects-1673504-supplementary.pdf]

I2I-SOP-004: Strain characterisation of resistant mosquitoes for monitoring bioefficacy in ITNs treated with two active ingredients (Dual-AI ITNs)

1. Purpose 2
2. Background 2
3. Materials & equipment 3
4. Procedure 4
   4.1. Test mosquitoes 4
   4.2. Tube bioassay setup 5
   4.3. Tube bioassay procedure 7
   4.3.1. Pyrethroid insecticide 7
   4.3.2. PBO synergism 9
   4.3.3. Non-pyrethroid AI 9
   4.4. Cone bioassay setup 9
   4.5. Cone bioassay procedure: new pyrethroid-only net 11
   4.6. Post-assay mosquito sample storage 12
5. Additional data collection 12
6. Deviations from standard protocol 12
7. Glossary of terms 13
8. References 13

1. Purpose

This standard operating procedure (SOP) describes the methods for characterising pyrethroid resistant mosquitoes used in bioefficacy testing with dual-AI-ITNs, primarily for durability monitoring studies. However, this SOP could be adapted for new types of ITNs, as they are developed, and for other types of studies requiring resistant mosquito strains. The process used to determine the methodology detailed in this SOP, and justifications for key methodological parameters can be found in 'Strain Characterisation for Measuring Bioefficacy of ITNs Treated with Two Active Ingredients (Dual-AI ITNs) for Durability Monitoring: Developing a Robust Protocol by Building Consensus' (Lees et al., 2022)

2. Background

Durability monitoring of insecticide-treated nets (ITNs) containing a pyrethroid in combination with a second active ingredient (AI) must be adapted so that the insecticidal bioefficacy of each AI can be monitored independently. An effective way to do this is to measure rapid knock down of pyrethroid-susceptible strain of mosquitoes to assess the bioefficacy of the pyrethroid component and a use pyrethroid-resistant strain to measure the bioefficacy of the second ingredient. To allow robust comparison of results across tests within and between test facilities and across time throughout a study, durability monitoring protocols must also include either characterisation of the strain, standardisation of the mosquitoes used for bioassays, or a combination of the two. This approach to characterise the mosquitoes used for bioefficacy testing was agreed by the consensus of a stakeholder group (Lees et al., 2022). The experimental methodology uses standard World Health Organization (WHO) tube assays (WHO, 2016) and cone tests (WHO, 2013) to evaluate the strains.

3. Materials & equipment

General

- Data collection sheets
- Lab coat
- Gloves
- Aspirator (manual/mechanical), separate for each insecticide
- Mosquito strains
- Pen/permanent markers
- Temperature and humidity data logger
- Timer
- 10% sucrose solution (e.g., sugar or honey and water)

Tube Test

- WHO tube test kits (number of tubes required listed below for each test)
    - Green dot holding tubes with steel clips
    - Red dot AI exposure tubes with copper clips
    - Yellow dot control exposure tubes with copper clips
    - Slider units

Cone test

- Pyrethroid treated test nets
- Control untreated net
- Paper labels
- Aluminium foil
- Tape
- Mosquito holding containers (e.g., paper cups covered with untreated netting held by elastic bands)
- Cone holding frame (x 2), with holes to hold standard WHO plastic cones
- Cone holder frame stand, which holds frame at 45°
- WHO plastic cones
- Binder clips or clamps
- Cotton wool or rubber stoppers
- Cotton wool

4. Procedure

- The resistant mosquito strain should be characterised before dual-AI ITN bioefficacy testing; for example, in a durability monitoring study where net samples are at 6- and 12-months post-distribution the characterisation should be repeated alongside sample testing at each time point. The characterisation should be conducted no more than one month before starting. If a round of bioassays takes longer than one month a round of bioassays it should be repeated within one month of finishing. The characterisation should also be repeated for longer studies – every mosquito generation if possible.
- To characterise the resistant strain the following bioassay will be conducted:
    - Pyrethroid WHO susceptibility tube bioassay (hereafter referred to as 'tube bioassay') (Section 4.3.1)
    - PBO synergism tube bioassay (Section 4.3.2)
    - Non-pyrethroid AI tube bioassay/bottle bioassay (Section 4.3.3)
    - New pyrethroid-only ITN WHO cone bioassay (hereafter referred to as 'cone bioassay') (Section 4.5)

4.1. Test mosquitoes

- Use 2-to-5-day-old non-blood fed female *Anopheles* mosquitoes originating from a laboratory colony.
- Standardised rearing and quality control of the colony of mosquitoes used for testing is recommended (Williams et al., 2019).
- Wing length measurements (Yeap et al., 2013) should be taken of the colony at the time of characterisation as an indication of size. When this is not possible dry weight can be used.
- Characterisation should be conducted on a resistant mosquito strain, and a susceptible mosquito strain (Table 1) included as a control.
- The number of mosquitoes required for each test is provided in Table 2.

Table S1. Characteristics of pyrethroid-susceptible strains (Lees et al, 2022)

Resistance status	Definition
Pyrethroid-susceptible	Mosquito mortality is >90% following exposure to the test pyrethroid's diagnostic concentration (DC) in both a standard WHO tube bioassay[1] and a WHO cone bioassay[2] using a new pyrethroid net.

[1] Test procedures for insecticide resistance monitoring in malaria vector mosquitoes (WHO, 2016); [2] Guidelines for laboratory and field-testing of long-lasting insecticidal nets (WHO, 2013)

Table S2. Numbers of 'resistant' test mosquitoes and susceptible mosquitoes required for strain characterisation by bioassay.

Assay	Strain	Treatment	Replicates	n per replicate	n total
Pyrethroid tube	Resistant	Pyrethroid	4	25	100
		Control	2	25	50
		Sub-total	150		
	Susceptible	Pyrethroid	1	25	25
		Control	1	25	25
		Sub-total	50		
PBO synergism tube	Resistant	Pyrethroid + PBO	4	25	100
		PBO - only	2	25	50
		Sub-total	150		
Non-pyrethroid AI tube/bottle	Resistant	Non-Pyrethroid	4	25	100
		Control	2	25	50
		Sub-total	150		
Pyrethroid net cone bioassay	Resistant	Pyrethroid	4	5	20
		Control	1	5	5
		Sub-total	25		
	Susceptible	Pyrethroid	4	5	20
		Control	1	5	5
		Sub-total	25		
**Total**	Resistant	475			
	Susceptible	75			
	Total	550			

4.2. Tube bioassay setup

4.2.1. Test papers

- Test papers can be purchased directly from WHO (WHO, 2016). Self-made test paper should be 12 X 15 cm and treated with the established standard discriminating concentration of insecticide (Table 3, (WHO, 2016)). Discriminating concentrations have recently been recommended for chlorfenapyr or pyriproxyfen (WHO, In Prep)

Table S3. AI discriminating concentrations for Anopheles, one-hour exposure in a WHO tube test (WHO, 2016)

**Insecticide**	***Anopheles* discriminating concentration (%)**
Alpha-cypermethrin	0.05
Deltamethrin	0.05
Permethrin	0.75
PBO	4%

4.2.2. Equipment setup

- Gloves and a lab coat should always be worn when handling test papers and should be changed between different papers with different AIs to avoid cross-contamination.
- Clean testing area and equipment as specified by the labs cleaning protocols.
- Test mosquitoes and insecticide papers should be acclimatised to the climatic conditions of the testing room for a minimum of one hour before testing.
- Prepare required WHO testing tube(s) with the same pyrethroid as in the net being assayed.
    - Tubes with green dots should be used for holding tubes. Tubes with a red-coloured dot should be used for insecticide-treated or synergist papers. Tubes with a yellow-coloured dot should be used for untreated control papers.
    - Place one paper inside one tube by rolling it into a cylinder and inserting it into the tube. Secure the paper inside the tube with a metal clip. For holding tubes (green dot) use steel clips; for control (yellow dot) and AI-treated (red dot) tubes, use copper clips.
    - Attach the slide unit to the green-dot holding tubes.
- Prepare the test mosquitoes (Table 2) by carefully transferring the required mosquitoes to holding tubes through the hole in the slide unit, 20-25 mosquitoes per tube using an aspirator. Place the holding tube in an upright position. Remove any knocked-down mosquitoes from holding tubes before testing.

4.3. Tube bioassay procedure

4.3.1. Pyrethroid insecticide

- Set up the equipment described above (section 4.2) with 8 holding tubes, five pyrethroid test tubes, and three untreated control tubes (Figure 1).
    - Resistant strain: Six holding tubes, four pyrethroid tubes, and two control tubes
    - Susceptible strain: Two holding tubes, one pyrethroid tube, and one control tube
- Record the temperature and humidity during testing. Preferably continuously with a data logger or manually at the start and end of exposure, and the end of the mosquito holding period.
- Expose mosquitoes to test and control papers for 1 hour:
    - Attach the treatment/control exposure tube to the holding tube by screwing it into the slide unit.
    - Carefully open the slide unit and blow through the mesh of the holding tube to transfer mosquitos from the holding tube into the exposure tube.
    - When all mosquitoes have been transferred into the exposure tube, carefully close the slide unit, and unscrew the holding tube. Leave holding tubes to one side.
    - Place the exposure tube vertically and leave for one hour.
    - After one hour, transfer the mosquitoes back to the holding tube using the same method (attach the holding tube to the slide unit, blow through the exposure tube mesh to move mosquito back to the holding tube).
    - Ensure holding tubes are correctly labelled with the test sample, test rep, mosquito species, and testing date.
- Provide mosquitoes with a sugar meal (10% sucrose solution soaked onto a relevant substrate such as cotton wool).
- Record the number of mosquitoes in each holding container to expose the total numbers.
- After 1-hour post-exposure, record the number of mosquitoes knocked down (Table *Table S4. The definitions used for classifying alive, knocked down or dead mosquitoes, adapted from*4).
- After 24-hours post-exposure, record the number of dead mosquitoes (Table *Table S4. The definitions used for classifying alive, knocked down or dead mosquitoes, adapted from*4).
- If the outcome for the non-pyrethroid AI being evaluated in the test study is mortality after more than 24-hours, extend the mortality scoring period in the pyrethroid AI tests to match, e.g. if the

second AI being tested is chlorfenapyr and mortality is counted until 72-hours, also record mortality in the pyrethroid tests to 72-hours.

- At the end of testing, ensure mosquitoes are stored correctly (see section 4.6.) for future analysis. If mosquitoes are not required for future analysis, discard mosquitoes safely.

Table S4. The definitions used for classifying alive, knocked down or dead mosquitoes, adapted from (WHO, 2016)

Mosquito status	Definition
Alive	The mosquito is mobile or able to stand or fly in a coordinated manner.
Knocked down	The mosquito is immobile or unable to stand or take off at 1-hour following net exposure.
Dead	The mosquito is immobile or unable to stand or take off at 24-hours (or as appropriate) following net exposure.

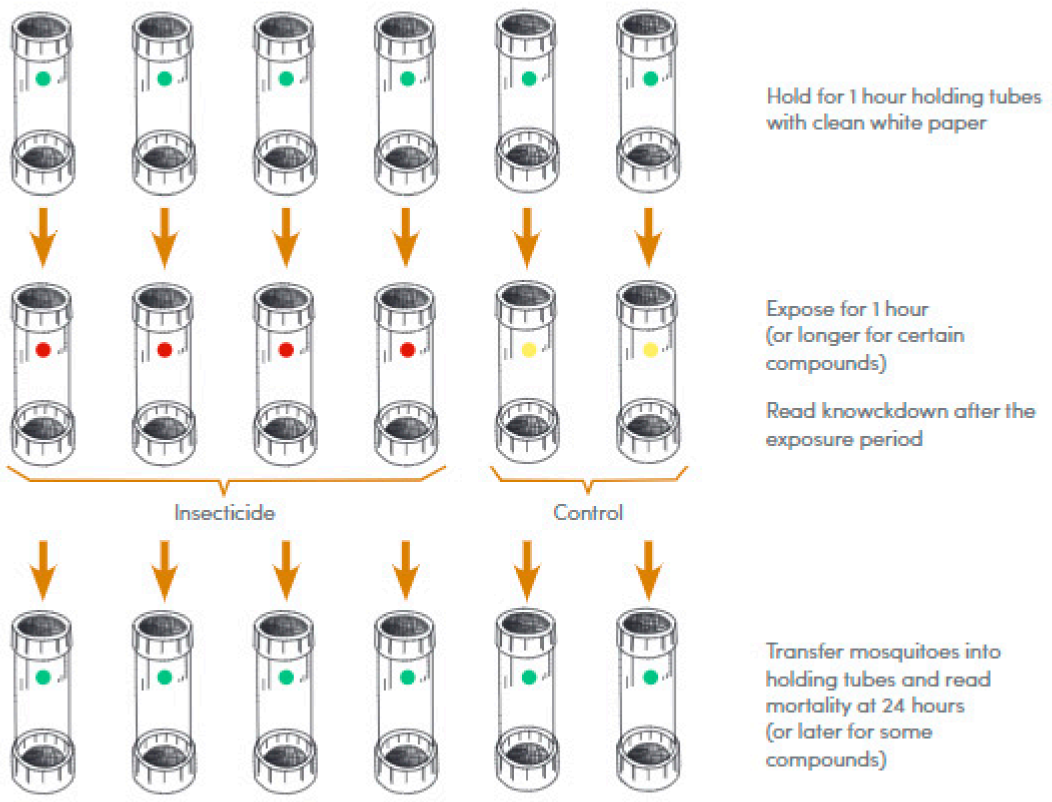

Figure S1. Infographic of testing to show tube bioassay setup, adapted from (WHO, 2016).

4.3.2. PBO synergism

- For the PBO synergism bioassay, the equipment setup and procedure are as described above (Section 4.3.1) with the following exceptions:
    - Set up the equipment as described above (section 4.2) with six holding tubes, four pyrethroid test tubes and six PBO-only tubes (no untreated control tube).
        - Only the resistant *Anopheles* strain is tested.
    - PBO Pre-exposure: Exposure mosquito to the PBO-only tubes using the exposure method outlined above (section 4.2) for one hour.
    - After this, exposure four of the tube replicates to the pyrethroid tubes using the same exposure method for one hour. The remaining two tubes are used as a PBO exposure only control.

4.3.3. Non-pyrethroid AI

- *Note: This step is not required for nets containing PBO as an additional active ingredient as susceptibility has already been confirmed previously in the PBO-synergism assay (Section 4.3.2).*
- *Note: For AIs without an established DC for treated papers a different appropriate method to test the strain against a DC for the second AI can be used i.e., CDC bottle bioassay* (Centers for Disease Control and Prevention, 2012).
- For the non-pyrethroid AI tube bioassay, the equipment setup and procedure are as described above (Section 4.3.1) with the following exceptions:
    - Only a resistant *Anopheles* strain is tested.
    - Negative control used depends on the AI; refer to the WHO guidelines (WHO, 2016)
    - Outcome measure depends on the AI; refer to the WHO guidelines (WHO, 2016)

4.4. Cone bioassay setup

4.4.1. Net Samples

- Gloves and a lab coat should always be worn when handling the nets and should be changed between handling nets/net panels with different AIs to avoid cross-contamination.
- New nets should be aired but unwashed. Air new nets away from direct sunlight for a minimum of 7-days before testing.

- The strain should be characterised with a new net of the same brand as tested for durability monitoring (Table 5).
- Hang net on a net frame. The net frame should be cleaned between nets as specified by the lab's cleaning protocols.
- Cut the net. Scissors should be changed or cleaned between cutting nets with different AIs.
    - For pyrethroid-only net: Cut 4 pieces (30 x 30 cm) from the net
    - For untreated control net: Cut 1 piece (30 x 30 cm) from the net
- Label net pieces with the sample position (i.e., 1 - 4) and net ID (e.g., Untreated 1) on paper labels secured to the corner of each piece.
- Wrap each piece individually in aluminium foil and refrigerate. If a refrigerator is not available, store nets in a cool, dry place at <5°C. Do not use net pieces >10 times. Cut new pieces as required.

Table S5. List of WHO prequalified (WHO, 2020) *pyrethroid-only nets categorised by pyrethroid.*

Pyrethroid	Brand Name	Manufacturer
Alpha-cypermethrin	DuraNet LN	Shobikaa Impex Private Limited
	Interceptor	BASF
	MAGNet	V.K.A. Polymers Pvt. Ltd
	MiraNet	A to Z Textile Mills Limited
	Royal Sentry	Disease Control Technology LLC
	Royal Sentry 2.0	Disease Control Technology LLC
	SafeNet	Mainpol GmbH
Deltamethrin	Panda Net 2.0	Life Ideas Biotechnology Co. Ltd
	PermaNet 2.0	Vestergaard Sarl
	Reliefnet Reverte	Real Relief Health ApS
	Tsara	Moon Netting FZCO
	Tsara Soft	Moon Netting FZCO
	Yahe LN	Fujian Yamei Industry & Trade Co. Ltd
	Yorkool LN	Tianjin Yorkool International Trading Co., Ltd
Permethrin	Olyset Net	Sumitomo Chemical Co., Ltd

4.4.2. Equipment setup

- Gloves and a lab coat should always be worn when handling the nets and should be changed between different nets with different AIs to avoid cross-contamination.
- Clean testing area and equipment as specified by the labs cleaning protocols.
- Prepare test mosquitoes (Table 2) by carefully transferring required mosquitoes to holding containers, 5 mosquitoes per container using an aspirator.
- Test mosquitoes and net samples should be acclimatised to the climatic conditions of the testing room for a minimum of one hour before testing. Remove any knocked-down mosquitoes from holding containers before testing.
- Prepare cone testing board(s).
  - Place 1st cone holder frame in the stand.
  - Secure control and test nets to 1st cone holder frame with tape. Ensure nets do not overlap to avoid cross-contamination, that they are correctly labelled, and that the labels are visible.
  - Place the plastic cones over the nets and secure the cones in place by placing the 2nd cone holder frame over the top. The two cone holder frames can be secured using binder clips or clamps.
  - Make sure that the board is stable and situated at a 45° angle.
  - Cover the opening of the plastic cones with a stopper (e.g., rubber plug or cotton wool).

4.5. Cone bioassay procedure: new pyrethroid-only net

- Record the temperature and humidity during testing. Preferably continuously with a data logger or manually at the start and end of exposure and the end of the mosquito holding period.
- Exposed batches of 5 mosquitoes to netting pieces for 3 minutes to test netting (4 replicates) or untreated control (1 replicate):
  - Remove the stopper from the cone and transfer 5 mosquitoes from the holding container into the plastic cone using an aspirator. Take care not to touch the net with the aspirator end resulting in contamination.
  - Cover the cone with the stopper to prevent mosquitoes from escaping.
  - Expose mosquitoes to the netting sample for 3 minutes.
  - Transfer mosquitoes from the cone back to their holding container with an aspirator. Take care not to touch the net with the aspirator end resulting in contamination. Ensure containers are correctly labelled with the net sample ID (Net ID and position), test rep, mosquito species, and testing date.
  - Repeat until all replicates have been exposed.

- Provide mosquitoes with a sugar meal (10% sucrose solution soaked onto a relevant substrate such as cotton wool).
- Record the number of mosquitoes in each holding container to expose the total numbers.
- After 1-hour post-exposure, record the number of mosquitoes knockdown (Table *Table S4. The definitions used for classifying alive, knocked down or dead mosquitoes, adapted from*4).
- After 24-hours post-exposure, record the number of dead mosquitoes (Table *Table S4. The definitions used for classifying alive, knocked down or dead mosquitoes, adapted from*4).
- If the outcome for the non-pyrethroid AI being evaluated in the test study is mortality after more than 24 hours, extend the mortality scoring period in the pyrethroid AI tests to match, e.g. if the second AI being tested is chlorfenapyr and mortality is counted until 72 hours, also record mortality in the pyrethroid tests to 72 hours.
- At the end of testing, ensure mosquitoes are stored correctly (see section 4.6.) for future analysis. If mosquitoes are not required for future analysis, discard mosquitoes safely.

4.6. Post-assay mosquito sample storage

- Negative control tubes and cones: Store all surviving mosquitoes of the resistant strain in pools of 5 mosquitoes in RNALater, for further characterisation if needed in future.
- AI test tubes and cones: Store all mosquitoes of the resistant strain, separated into those that were killed and those that survived, *in silica*.

5. Additional data collection

- Measure the wing length of the stored samples and report average lengths alongside the characterisation results.
- Where resources allow additional fitness indices such as dry weight or wing symmetry can also be measured.
- The samples stored *in silica* can also be used for further molecular analysis if any is required after molecular characterisation of the samples stored in RNALater.

6. Deviations from standard protocol

The resistant mosquitoes used for bioefficacy testing of dual-AI ITNs should ideally originate from a stable laboratory colony, maintained under standard conditions and subject to quality control

measures, to minimise changes in phenotype over time. F_0 adults collected from larval breeding sites should only be used when a lab strain is unavailable.

7. Glossary of terms

AI	Active ingredient
Cone bioassay	A WHO cone bioassay
CDC	Centers for Disease Control and Prevention
DC	Diagnostic concentration
Dual-AI ITN	An insecticide-treated net treated with two active ingredients
I2I	Innovation 2 Impact
ITN	Insecticide-treated net
PBO	Piperonyl Butoxide
SOP	Standard Operating Procedure
Tube Bioassay	A WHO susceptibility tube bioassay
WHO	World Health Organization

8. References

Centers for Disease Control and Prevention, (2012) Guideline for Evaluating Insecticide Resistance in Vectors Using the CDC Bottle Bioassay. *CDC Methods*.

Lees, R., (2022) Strain Characterisation for Monitoring Durability of Bioefficacy in ITNs Treated with Two Active Ingredients (Dual-AI ITNs): Developing a Robust Protocol by Building Consensus. *Insects*.

WHO, (2013) *Guidelines for laboratory and field-testing of long-lasting insecticidal nets. WHO/HTM/NTD/WHOPES/20131*. Geneva: World Health Organization.

WHO, (2016) *Test procedures for insecticide resistance monitoring in malaria vector mosquitoes*. Geneva: World Health Organization.

WHO, (2020) *Prequalified Vector Control Products | WHO - Prequalification of Medical Products (IVDs, Medicines, Vaccines and Immunization Devices, Vector Control)*. [online] Available at: https://extranet.who.int/pqweb/vector-control-products/prequalified-product-list [Accessed 2 Aug. 2021].

WHO, (2022) *Determining discriminating concentrations of insecticides for monitoring resistance in mosquitoes: report of a multi-centre laboratory study and WHO expert consultations.* Geneva.

Williams, J., Flood, L., Praulins, G., Ingham, V.A., Morgan, J., Lees, R.S. and Ranson, H., (2019) Characterisation of Anopheles strains used for laboratory screening of new vector control products. *Parasites and Vectors*.

Yeap, H.L., Endersby, N.M., Johnson, P.H., Ritchie, S.A. and Hoffmann, A.A., (2013) Body Size and Wing Shape Measurements as Quality Indicators of Aedes aegypti Mosquitoes Destined for Field Release. *The American Journal of Tropical Medicine and Hygiene*, [online] 891, p.78. Available at: /pmc/articles/PMC3748492/ [Accessed 16 Feb. 2022].
